# Prescription patterns of sacubitril/valsartan among patients with hypertension in Japan

**DOI:** 10.3389/fcvm.2026.1738988

**Published:** 2026-07-17

**Authors:** Aya F. Ozaki, Hisanaga Nomura, Tomohiro Terada, Eri T. Kato, Ali A. Naqvi, Julie T. Nguyen, Yuki Kunitsu

**Affiliations:** 1 Department of Clinical Pharmacy Practice, School of Pharmacy and Pharmaceutical Sciences, University of California, Irvine, CA, United States; 2 Department of Clinical Pharmacology and Therapeutics, Kyoto University Hospital, Kyoto, Japan; 3 Department of Cardiovascular Medicine, Kyoto University Hospital, Kyoto, Japan; 4 Mary & Steve Wen Cardiovascular Division, University of California Irvine Health, Orange, CA, United States

**Keywords:** hypertension, Japan, prescription patterns, real-world data, sacubitril/valsartan

## Abstract

**Background:**

Sacubitril/valsartan (S/V) is an established therapy for heart failure that has been recently approved for hypertension in Japan. However, evidence regarding its use in combination with other antihypertensive agents in real-world practice is limited.

**Objective:**

To describe real-world prescribing patterns of S/V among patients with hypertension in Japan.

**Methods:**

A retrospective cohort study using a nationwide Japanese electronic health record database (August 2020 to October 2023) identified adults with hypertension (ICD-10: I10–I15) who were newly prescribed S/V. Patients with heart failure were excluded. Concomitant antihypertensive medications, S/V dosing, and discontinuation patterns were assessed over 90 days following initiation.

**Results:**

Of the 9,486 patients (median age 79 years; 50.6% men), 63.6% received S/V with a calcium channel blocker (CCB), 22.5% as monotherapy, and 11.8% as triple therapy with CCB and thiazide. At day 90, 55.5% of patients received S/V plus a CCB, and 26.0% were on S/V monotherapy. The S/V discontinuation rate reached 11.8% by day 90. Approximately half of those on triple therapy discontinued the thiazide diuretic within 30 days.

**Conclusions:**

In Japanese patients with hypertension, S/V is most commonly used in combination with a CCB, although a notable proportion receive it as monotherapy. These findings highlight the need for further studies to evaluate the safety, efficacy, and cost-effectiveness of S/V-based combination regimens.

## Introduction

Sacubitril/valsartan (S/V) is a well-established therapy for heart failure with reduced ejection fraction, supported by strong evidence and integrated into international guideline recommendations (1). Beyond its cardioprotective role, S/V has demonstrated significant blood pressure (BP)-lowering effects, leading to its approval for the treatment of essential hypertension in several Asian countries, including Japan (2). Notably, whereas S/V is used predominantly for heart failure in Western countries, the Japanese Ministry of Health, Labour and Welfare formally approved it for the treatment of essential hypertension. Consequently, its real-world utilization patterns in patients without heart failure, for whom it is specifically prescribed for its antihypertensive properties, warrant dedicated investigation (2). However, current randomized controlled trials (RCTs) in hypertension have predominantly compared S/V with angiotensin receptor blockers (ARBs), often without permitting concomitant antihypertensive therapy, thereby limiting available evidence on the safety and efficacy of S/V in combination regimens for this population (3). Furthermore, although current hypertension guidelines and the S/V package insert in Japan do not recommend S/V as a first-line agent, they provide limited guidance on optimizing first-line agents before S/V initiation (3). Given these evidence gaps, we conducted a real-world analysis to evaluate the prescribing patterns of S/V in combination with other antihypertensive agents among patients with hypertension using a large-scale Japanese electronic health record (EHR) database.

## Methods

We conducted a retrospective cohort study using a nationwide EHR database in Japan between 1 August 2020 and 31 October 2023. Adults aged ≥20 years with a diagnosis of hypertension (ICD-10: I10–I15) who were newly prescribed S/V were included. Patients with a history of heart failure (HF) or who received an HF diagnosis within 90 days of S/V initiation, or who could not be followed up for 1 year prior to and 90 days after S/V initiation, were excluded. These exclusions were applied to isolate the prescribing patterns of S/V strictly for its hypertension indication, ensuring that the cohort reflected patients receiving the drug for its antihypertensive properties rather than for heart failure. We assessed antihypertensive medication use during the 30 days before and 90 days after S/V initiation. The medication classes examined included renin–angiotensin–aldosterone system (RAAS) inhibitors, calcium channel blockers (CCBs), and thiazide diuretics. We also examined S/V dosing and discontinuation patterns during the 90-day follow-up period.

## Results

A total of 9,486 patients with hypertension were initiated on S/V during the study period. The median age was 79 years, and 50.6% were men. Comorbid conditions included diabetes in 15.6% of patients, prior myocardial infarction in 3.2%, and cancer in 13.3%.

**Figure 1:**
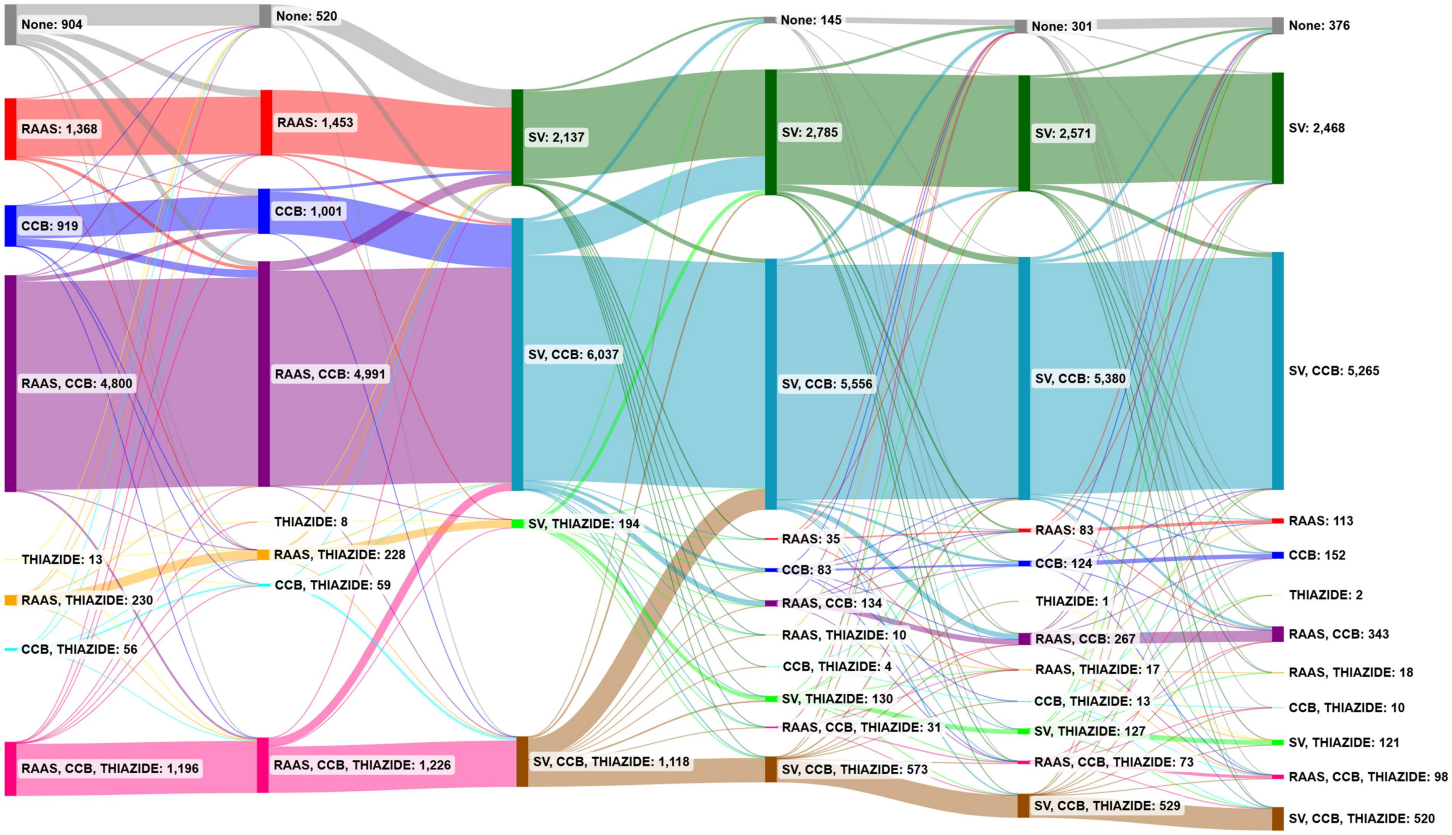
A Sankey diagram illustrating transitions in antihypertensive therapy before and after the initiation of sacubitril/valsartan (S/V) among patients with hypertension in Japan. CCB, calcium channel blocker; RAAS, renin–angiotensin–aldosterone system inhibitor; SV, sacubitril/valsartan.

At 14 days before S/V initiation, 5.5% of patients were not receiving any antihypertensive therapy. Monotherapy was used by 26.0% of the patients, while 55.6% received dual therapy. The most common dual regimen was an RAAS inhibitor plus a calcium channel blocker (CCB) (52.6%), followed by an RAAS plus thiazide (2.4%) and thiazide plus CCB (0.6%). Triple therapy was used in 12.9% of patients. Overall, 83.3% of patients were treated with RAAS inhibitors prior to S/V initiation (Figure 1).

At the time of S/V initiation, 63.6% of patients received S/V in combination with a CCB, 22.5% received S/V as monotherapy, and 11.8% were treated with S/V plus a CCB and thiazide triple combination therapy. By day 90, 55.5% were on S/V plus a CCB, 26.0% were on S/V monotherapy, and 5.5% were on a triple therapy regimen. S/V was discontinued in 4.6% of patients by day 30, 9.3% by day 60, and 11.8% by day 90. Regarding initial S/V dosing, 37.5% of patients received ≤100 mg/day, 62.3% received 150–200 mg/day, and 0.2% received >200 mg/day. At day 90, 26.0% of patients received ≤100 mg/day, while 53.6% received ≥200 mg/day.

## Discussion

Among patients with hypertension who were initiated on S/V, nearly half transitioned from a RAAS inhibitor plus CCB to an S/V plus CCB, with the majority (75.6%) maintaining this combination through to day 90. Despite the existing evidence gap regarding the use of S/V in combination with other antihypertensive agents, our findings demonstrated that dual combination therapy with S/V is common in real-world practice. However, our study identified potential concerns with triple combination therapy involving S/V, a CCB, and a thiazide diuretic, as approximately half of these patients discontinued the thiazide diuretic by day 30 and were continued on S/V plus CCB therapy. Given that sacubitril has inherent diuretic properties, combining it with a thiazide diuretic could increase the risk of side effects, potentially contributing to early thiazide discontinuation. These findings highlight the need for further research to better characterize the safety and tolerability of S/V-based combination therapies in patients with hypertension.

Nearly one-third of patients in our study were initiated on S/V without transitioning from multiple first-line antihypertensive agents, and approximately one-quarter received S/V as monotherapy by day 90. Because combination therapy with agents with different mechanisms is commonly used to achieve adequate BP control, S/V, which combines two agents with complementary mechanisms, may offer effective BP reduction while minimizing pill burden, thus presenting a practical advantage. However, while RCTs for hypertension have demonstrated the superiority of S/V over ARBs, evidence comparing S/V with other antihypertensive classes or combination therapies remains limited, with one trial demonstrating non-inferiority to CCBs (4). Widespread use of S/V without prior use of multiple first-line antihypertensive agents raises financial concerns, particularly considering its higher cost relative to generic first-line agents. In the context of limited efficacy evidence for different antihypertensive agents and combination regimens involving S/V, along with substantial variation in S/V costs across countries, and a lack of cost-effectiveness analyses specific to hypertension management in Japan, further studies are needed to evaluate both the comparative efficacy and the economic impact of S/V.

This study has several limitations. It is a retrospective analysis and was conducted using data from a Japanese population, which may limit its generalizability to other populations with different prescribing practices, drug availability, or reimbursement structures. The dataset did not include information on the clinical rationale for medication selection. Additionally, unmeasured confounders such as hypertension severity, lifestyle factors, and physician and patient preferences may have influenced prescribing patterns.

## Conclusion

In this real-world analysis of patients with hypertension without HF in Japan, the majority of subjects received S/V in combination with a CCB, while one-quarter received S/V as monotherapy. Given the limited evidence regarding the efficacy, safety, and cost-effectiveness of S/V combination therapies, the judicious use of established first-line antihypertensive agents and the careful selection of S/V-based combination regimens remain essential. Our findings highlight the need for further research to establish evidence-based prescribing strategies and inform clinical practice and policy decisions in the management of hypertension.

## Data Availability

The datasets presented in this article are not readily available because they are under license for the current study, and hence are not publicly available. Data are, however, available from the authors upon reasonable request and with the permission of DeSC Healthcare Inc. Requests to access the datasets should be directed to the corresponding author.
